# Analysis of Core–Periphery Structure Based on Clustering Aggregation in the NFT Transfer Network

**DOI:** 10.3390/e27040342

**Published:** 2025-03-26

**Authors:** Zijuan Chen, Jianyong Yu, Yulong Wang, Jinfang Xie

**Affiliations:** School of Computer Science and Engineering, Hunan University of Science and Technology, Xiangtan 411100, China; chenhaha4086@163.com (Z.C.); wyl@mail.hnust.edu.cn (Y.W.); fifthxie@outlook.com (J.X.)

**Keywords:** Ethereum, NFT, network structure, core–periphery structure, random block model, cluster aggregation

## Abstract

With the rise of blockchain technology and the Ethereum platform, non-fungible tokens (NFTs) have emerged as a new class of digital assets. The NFT transfer network exhibits core–periphery structures derived from different partitioning methods, leading to local discrepancies and global diversity. We propose a core–periphery structure characterization method based on Bayesian and stochastic block models (SBMs). This method incorporates prior knowledge to improve the fit of core–periphery structures obtained from various partitioning methods. Additionally, we introduce a locally weighted core–periphery structure aggregation (LWCSA) scheme, which determines local aggregation weights using the minimum description length (MDL) principle. This approach results in a more accurate and representative core–periphery structure. The experimental results indicate that core nodes in the NFT transfer network constitute approximately 2.3–5% of all nodes. Compared to baseline methods, our approach improves the normalized mutual information (NMI) index by 6–10%, demonstrating enhanced structural representation. This study provides a theoretical foundation for further analysis of the NFT market.

## 1. Introduction

Blockchain technology was first developed as the foundation for Bitcoin. Due to its success, enterprises and financial institutions began exploring its potential. Ethereum, an extension of blockchain technology, enables asset creation and trading via smart contracts. This includes Ether, ERC-20 tokens, and non-fungible tokens (NFTs). NFTs are unique digital assets with traceable ownership and transaction histories recorded on the blockchain. Since 2021, the NFT market has experienced rapid growth. However, existing studies lack a systematic understanding of NFT transfer patterns.

Modeling NFT transfers as a network reveals critical structures like the core–periphery configuration. This can inform market regulation and business models. Existing studies oversimplify NFT networks by assuming binary core–periphery roles [1]. They ignore layered interactions between creators, traders, and platforms. Our work addresses this gap through probabilistic modeling.

In network science, the core–periphery structure categorizes nodes into a dense core and a sparse periphery [2]. Classical methods like k-core decomposition [3] identify nested layers but fail to encode domain-specific knowledge (e.g., NFT transaction semantics). We propose a Bayesian stochastic block model (SBM) to address this. It incorporates prior knowledge to extract hub-and-spoke and layered core–periphery structures.

We also introduce a cluster aggregation framework. It unifies diverse core–periphery partitions by weighting them via MDL and variation in information. Our experiments show that our aggregated structures preserve richer features than individual methods.

The main contributions of this study are as follows:

- We propose a core–periphery stochastic block model that incorporates prior knowledge. This leads to deriving the hub-and-spoke and layered models, which extract core–periphery structures in the NFT transfer network.
- We employed the model’s MDL to evaluate different core–periphery structures and, based on the evaluation results, determine local aggregation weights for the core–periphery aggregation process.
- We propose an integration-driven clustering metric combined with the fit of core–periphery structures and a locally weighted core–periphery structure aggregation scheme. This scheme balances the diversity and local reliability of core–periphery structures.

The remainder of this paper is organized as follows. Section 2 reviews the structural analysis of the NFT transfer network, research on core–periphery structures, and related work on cluster aggregation. Section 3 elaborates on the proposed method. The experimental results are presented in Section 4. Finally, we conclude the paper in Section 5.

## 2. Related Work

With the rise of Ethereum, research on blockchain transaction networks has expanded rapidly. Chen et al. [4] conducted one of the first graph-based analyses of Ethereum transactions. Their study revealed a hierarchical structure where only 10% of traders control 80% of the transaction volume. Similarly, Bartoletti et al. [5] analyzed over 1000 smart contracts and uncovered significant design flaws in decentralized exchanges. Ante et al. [6] mapped CryptoPunks and Art Blocks transaction graphs for NFTs. They found that the top 5% of traders influence 60% of NFT liquidity. However, systematic studies on NFT transfer networks—especially core–periphery structures—remain rare. Preliminary works [7,8] focus on price prediction but ignore structural roles. For example, ref. [7] uses ML models to predict NFT prices without analyzing trader hierarchy—a gap our work addresses. While link prediction methods [9] using random-walk and maximum likelihood approaches effectively reconstruct network structures, their application to NFT transaction dynamics—particularly in modeling role-aware topological evolution—remains unexplored.

Core–periphery theory, rooted in economic sociology [2], divides networks into dense cores and sparse peripheries. Borgatti’s two-block model [1] assumes binary roles: nodes are either core (highly connected) or periphery. In contrast, k-core decomposition [3] identifies nested layers. For instance, a three core contains all nodes with ≥3 connections, revealing a deeper hierarchy. Bayesian SBMs [10,11] add flexibility by encoding prior knowledge (e.g., “platforms as hubs”). However, these methods have limitations. The two-block model oversimplifies NFT networks. For example, it cannot distinguish between marketplaces (core hubs) and whale traders (secondary cores). SBMs lack metrics to evaluate financial semantics. A partition may fit structurally but misalign with trader roles (e.g., misclassifying creators as periphery). Beyond technical network analyses, Franceschet and Colavizza et al. [12] demonstrated how polycentric governance in crypto art ecosystems—through decentralized authorship models involving artists, collectors and scholars—necessitates new analytical frameworks that reconcile transactional hierarchies with stakeholder role semantics.

Clustering aggregation methods aim to unify partitions. Yu et al. [13] proposed pairwise consensus, where nodes co-occurring across partitions are grouped. Huang et al. [14] transformed aggregation into hypergraph cuts, optimizing global consensus. For overlapping communities, ref. [15] extended metrics like NMI to handle overlaps. Yet, most methods [13,14,16] weigh all partitions equally. This ignores quality differences—a flawed assumption in NFT networks wherein methods like k-core [3] may oversimplify these networks. Xie et al. [17] introduced MDL to measure partition quality. MDL selects models that balance fitness and complexity. While effective in social networks [17], MDL has not been adapted to financial networks where metadata (e.g., trader types) must align with partitions. Our framework fills this gap by integrating MDL with NFT-specific semantics.

## 3. Methods

**Figure 1:**
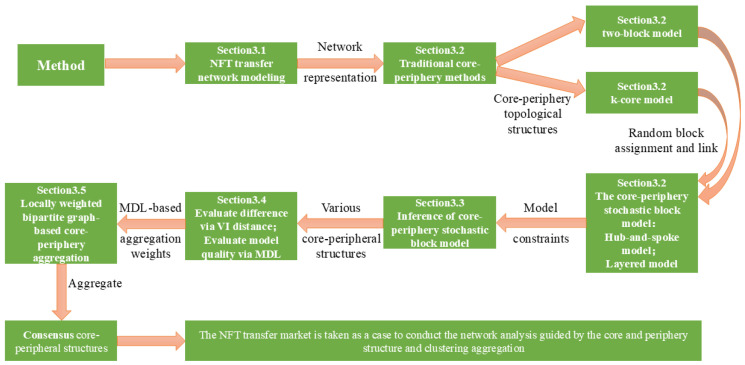
Method framework diagram.

This section systematically introduces the proposed Bayesian stochastic block model and the locally weighted core–periphery structure aggregation scheme (LWCSA). The process is divided into four steps: (1) core–periphery structure extraction using the stochastic block model, (2) evaluation of different core–periphery structures via the minimum description length (MDL) model, (3) determination of local aggregation weights, and (4) final aggregation using LWCSA. The overall framework is illustrated in Figure 1.

### 3.1. NFT Transfer Network

**Figure 2:**
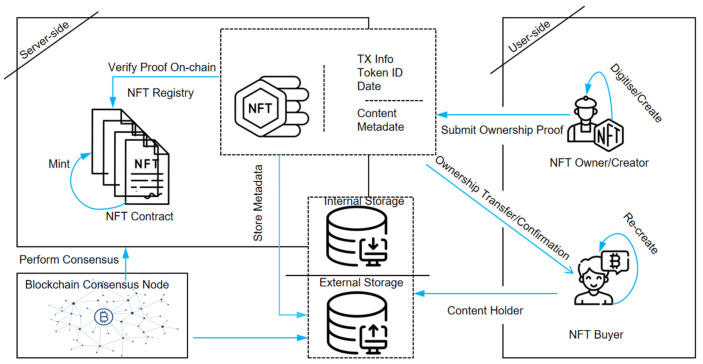
NFT transaction process.

NFT transactions rely on smart contracts, automating operations and storing transaction records transparently on the blockchain. The ERC-721 standard [7] defines the smart contract functionality required for NFT transactions, ensuring consistency in how NFTs are created, transferred, and tracked. A typical NFT transaction involves two key actions: the seller invokes the smart contract to transfer NFT ownership to the buyer. In contrast, simultaneously, the buyer’s account balance is transferred to the seller. Figure 2 illustrates this process. Each transaction can be modeled as a directed network edge, where the seller represents the source node, the buyer is the target node, and the transaction details (e.g., timestamp, price) are encoded as edge attributes. Modeling NFT transfers as a network enables researchers to analyze market trends, detect trading risks (e.g., wash trading, fraud), and optimize platform strategies.

Based on the transfer data of NFTs, we define the NFT transfer network. The NFT transfer network $G_{NFT}(V,E)$ consists of N nodes and M-directed edges without weights. Here, $V=\{v_{1},v_{2},\ldots ,v_{N}\},E=\{e_{ij}|i,j\in N\}$ represents the specific transfer record of an NFT. We use an adjacency matrix, $A_{NFT}={\left\{a_{ij}\right\}}_{N\times N}$, to represent the topological structure of the NFT transfer network, where $a_{ij}=1$ indicates a connection from node i to node j (i.e., the NFT seller transferring the NFT to the NFT buyer). Otherwise, $a_{ij}=0$.

Unlike dynamic networks, where edges evolve continuously, our analysis focuses on a static representation of the NFT transfer network. This static network aggregates all observed transactions within a predefined time window, capturing long-term structural relationships between traders rather than short-term fluctuations. While edge weights could reflect transaction frequency, our initial core–periphery detection phase adopts an unweighted approach to emphasize topological structure rather than trading intensity. However, in the later clustering aggregation stage, we introduce local weighting mechanisms to refine the final core–periphery structures by adjusting for structural uncertainties and improving aggregation consistency.

### 3.2. Core–Periphery Stochastic Block Models

Borgatti and Everett first introduced the core–periphery structure concept in 1999. Subsequent research has further developed and refined it [1]. Core nodes are typically necessary network connectors, often possessing higher degrees of betweenness centrality. In contrast, peripheral nodes have fewer connections and lower status. This structural concept is crucial for understanding two key network dynamics: information dissemination and influence propagation.

The stochastic block model (SBM) is a probabilistic graph model with broad applications. It is primarily used in community detection, social sciences, and bioinformatics. The model enables node partitioning into distinct blocks, allowing researchers to infer network structures through inter-group connection probabilities. The SBM proves particularly effective for identifying hierarchical structures like the core–periphery arrangement. This effectiveness stems from its ability to capture differential connection patterns between core and peripheral nodes. Our study uses the SBM to extract core–periphery structures in the NFT transfer network. We infer the probabilistic connections between core and peripheral nodes through this model.

Our NFT network analysis modeled the transfer network as a static directed graph. Nodes represent individual traders, while directed edges represent NFT transactions. Although NFT transactions inherently occur over time, our focus remains on the cumulative network structure. This approach better reveals the long-term hierarchical relationships between core and peripheral nodes. The stochastic block model aligns well with this static representation. Its strength lies in identifying probabilistic connections based on node roles (core or periphery).

The stochastic block model initially selects n nodes and randomly assigns these n nodes to several sets. Taking the example of using the stochastic block model in core–periphery partitioning, the selected nodes are randomly allocated to the core node set, ${set}_{core}$, and the periphery node set, ${set}_{pi}$. Each node has a probability, $p_{core}$, of being assigned to ${set}_{core}$. Correspondingly, the likelihood of being assigned to ${set}_{pi}$ is $p_{pi}=1-p_{core}$. Next, the model generates edges between nodes. An undirected edge is placed between each pair of nodes with a probability, $p_{st}$. Here, s and t represent the two nodes. Thus, the connection probability depends entirely on their set assignments. The probability matrix p_st_ is a 2 × 2 matrix in core–periphery partitioning. The matrix contains four probabilities, labeled as $p_{11}$, $p_{12}$, $p_{21}$, and $p_{22}$. Because undirected edges are placed, $p_{12}=p_{21}$, so only the probabilities $p_{11}$, $p_{12}$, and $p_{22}$ need to be considered.

**Figure 3:**
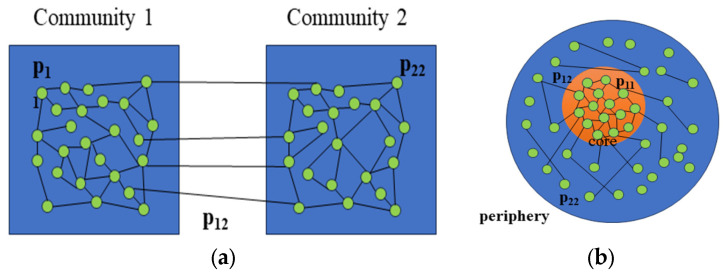
(**a**) SBM for community detection. (**b**) SBM for core–periphery structure.

In traditional stochastic block models used for community detection, higher within-block connectivity and lower between-block connectivity are considered. These conditions can be expressed as $p_{11}>p_{12}$ and $p_{22}>p_{12}$. However, when applying the stochastic block model to represent core–periphery structures, we can obtain $p_{11}>p_{12}>p_{22}$. In core–periphery structures, nodes assigned to ${set}_{1}$ are considered core nodes, while peripheral nodes are those in ${set}_{2}$, i.e., $p_{11}>p_{22}$. Additionally, the characteristics of core–periphery structures determine that connections between peripheral nodes are less likely than connections between peripheral nodes and core nodes, i.e., $p_{12}>p_{22}$. The different representations of community structures and core–periphery structures by the stochastic block model are illustrated in Figure 3.

The SBM is a widely used statistical model for network structure analysis. In this study, we apply the SBM to the NFT transfer network to extract its core–periphery structure. This approach forms the foundation of our proposed core–periphery stochastic block model, which can be a general statistical model for analyzing network structures.

The core–periphery stochastic block model operates on a key assumption: core and peripheral nodes are assigned to distinct blocks. In the context of the NFT transfer network, we assume there are N nodes with an adjacency matrix, $A_{N\times N}$. These N nodes are randomly allocated to M blocks. The block allocation status of nodes in the NFT transfer network is represented by vector D of length N, where $D=\{D_{1},D_{2},\ldots ,D_{N}\}$, and node $v_{i}$ assigned to block s is denoted as $D_{i}=s$. The collection probability between any two nodes is defined by an M × M matrix R. Here, $R_{st}$ represents the probability of a node in block s connecting to another node in block t. Through the modeling process, we observed that the core–periphery stochastic block model determines node connections based on their assigned blocks. Matrix R serves as the block connectivity matrix. While block allocation vector D and block connectivity matrix R are initially unknown in the NFT transfer network, their joint posterior distribution, $P\left(D,R|A\right)$, is essential for core–periphery partitioning. Based on Bayesian methods, we relate $P\left(D,R|A\right)$ to two prior probabilities, which leads to the following Equation (1). Here, ∝ denotes proportionality, and in specific statistical inference processes, this proportionality will be adjusted based on prior knowledge and evidence factors.

(1) P(D,R|A)∝P(D)P(R)P(A|D,R)

The core–periphery stochastic block model examines the core–periphery structure through node connections. These connections are reflected in block connectivity probability P(R). In the NFT transfer network, we only consider the block connectivity probability under specific block allocations. We integrate the two-block model and k-core decomposition method with the stochastic block model. This integration yields two types of core–periphery stochastic block models: the hub-and-spoke model and the layered model. The hub-and-spoke model divides network nodes into core and peripheral node sets. Core nodes are connected, and they are connected to some peripheral nodes, but peripheral nodes are not connected. The layered model employs k-core decomposition to partition nodes into hierarchical shells, where each layer reflects distinct structural and functional roles. In our model, layers are defined from the innermost core (Layer 1) to the outermost periphery (Layer L), with the probability of connections decreasing as we move outward. The roles of different layers are as follows:

Layer 1 (core) comprises high-value traders, major NFT platforms, and influential participants who drive market trends. These nodes are densely connected and handle the majority of transactions.

Layer 2 (secondary core) serves as a bridge between the core and outer layers. This layer includes active traders who frequently interact with core participants but have fewer direct connections.

Intermediate layers (3~l−1) represent traders with moderate transaction activity. These nodes contribute to market liquidity but do not exhibit strong influence individually.

Layer l (Periphery) consists of occasional traders, newcomers, and dormant accounts. These nodes have minimal connectivity and limited impact on the network’s structure.

**Figure 4:**
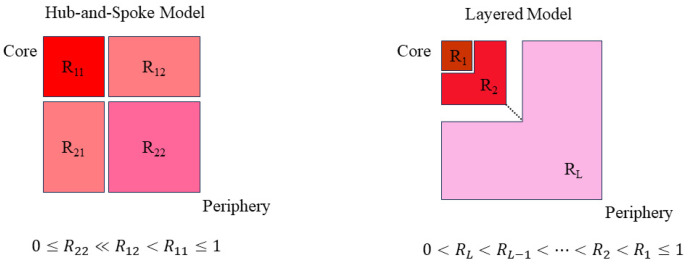
The topology of the hub-and-spoke model and layered model.

The connectivity probability matrix R follows $R_{1}>R_{2}>\ldots >R_{l}$, ensuring a structural hierarchy where core–layer interactions dominate. This framework captures multi-level market dynamics more effectively than binary core–periphery models. Schematic diagrams of these two models are shown in Figure 4. By applying Bayesian methods to the stochastic block model, we update the prior $P\left(R\right)$. This embeds different node allocation and core–periphery partitioning methods into the model, enabling statistical inference and model fitting.

*A:*  *Hub-and-spoke model*

The hub-and-spoke model fits the two-block model. In this model, the network is partitioned into fixed blocks: the core block and the peripheral block. The core block is encoded as ${block}_{1}$, while the peripheral block is encoded as ${block}_{2}$. From the definition of the two-block model, the definition of the hub-and-spoke model can be derived, where $R_{11}>R_{12}>R_{22}$ and $R_{11}=1,R_{12}=\alpha ,R_{22}=\beta$(1≥α≫β≥0). The definition establishes a clear core–periphery structure. The core is moderately or fully connected to the periphery. In contrast, connections between periphery nodes are minimal or nonexistent. Therefore, we constrain all prior probabilities of block connectivity matrix R of the hub-and-spoke model according to Equations (2) and (3).

(2) $${const}_{hub-and-spoke}(R)=\left\{\begin{array}{l} 1,\;\mathrm{if}\;0\leq R_{22}\ll R_{12}<R_{11}\leq 1\\ 0,\;otherwise \end{array}\right.$$

(3) $$P(R)\propto const_{hub-and-spoke}(R)$$

*B:*  *Layered model*

We assume the layered model consists of l layers. Here, layers correspond to the number of blocks used for node allocation in the stochastic block model. According to the k-core decomposition method definition, the probability of node connections gradually decreases from the innermost layer to the outermost layer. In the layered model, nodes in the innermost first layer are highly likely to be connected to nodes in other layers. However, the probability of nodes connecting to more peripheral layers decreases as we move outward. The node connection probability is further reflected in block connections. The degree of block connectivity decreases from the first layer to more peripheral layers. We describe the connectivity of block l as $R_{l}$. Based on this structure, we constrain all prior probabilities of block connectivity matrix R for the layered model. These constraints are defined in Equations (4) and (5).

(4) $$const_{layered}(R)=\left\{\begin{array}{l} 1,\;\mathrm{if}\;0<R_{l}<R_{l-1}<\ldots <R_{2}<R_{1}\leq 1\\ 0,\;otherwise \end{array}\right.$$

(5) $$P(R)\propto const_{layered}(R)$$

### 3.3. Inference of Core–Periphery Stochastic Block Structure

**Figure 5:**
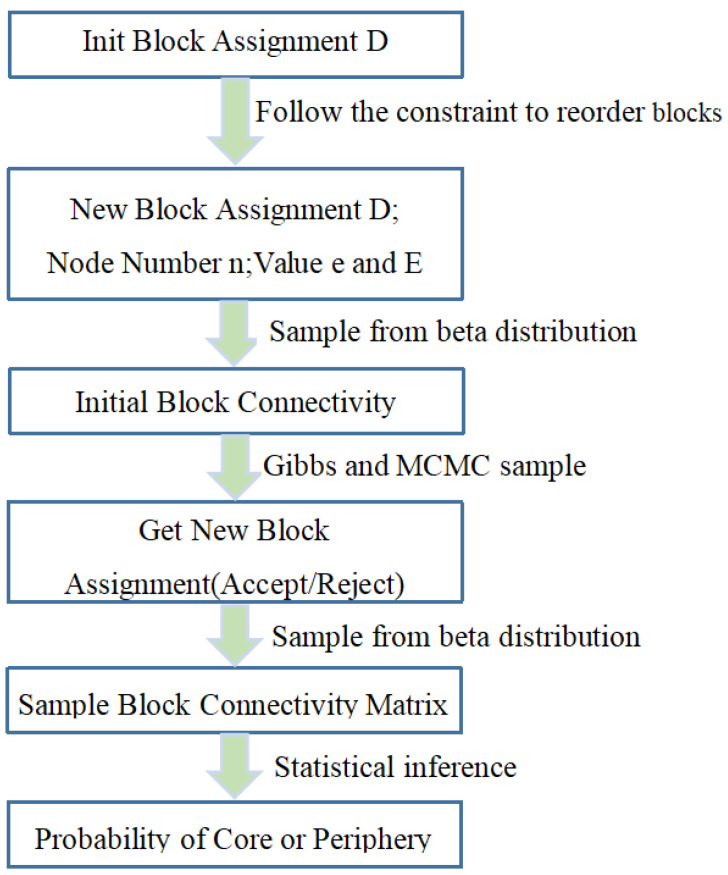
A process diagram of the core–periphery stochastic block model.

We propose two core–periphery stochastic block models. These models require statistical inference of two key components: block assignment vector D and block connection matrix R. The inference process for the core–periphery stochastic block models is illustrated in Figure 5. We implement Gibbs sampling to infer the distributions of D and R. Specific sampling steps are designed to collect samples based on the joint posterior distribution P(D,R|A). First, following the Gibbs sampling approach, we alternately sample D and R. Initially, we fix D and update R. Then, we fix the updated R and further update D. In the Gibbs sampling process, we assign the block s, to which ${node}_{i}$ is most frequently assigned, as the statistical block assignment, denoted as $\hat{D_{i}}=s$ Through this method, we can obtain the general joint posterior distribution P(D,R|A) in various network structures. To demonstrate the process, we take the Bayesian inference of the layered model as an example. The steps are detailed as follows:

First, in the sampling process of P(R|D,A), we focus on two blocks: ${block}_{s}$ and ${block}_{t}$. Then, we define two quantities: the actual number of edges $e_{st}$ between ${block}_{s}$ and ${block}_{t}$, and the maximum possible number of edges $E_{st}$ between them. Let $n_{s}$ and $n_{t}$ be the numbers of nodes in ${block}_{s}$ and ${block}_{t}$, respectively. Then, $E_{st}$ can be represented by Equation (6):

(6) $$Est=\left\{\begin{array}{ll} nsnt, & if\;s\neq t\\ \frac{ns(ns-1)}{2}, & otherwise \end{array}\right.$$

Next, we derive two expected values from $e_{st}$ and $E_{st}$. The first is $Y_{s}$, representing the expected number of edges originating from ${block}_{s}$ to other blocks. The second is $N_{s}$, representing the expected number of non-edges. These values are computed through the formulas $Y_{s}=\sum_{t\leq s}e_{st}$ and $N_{s}=\sum_{t\leq s}E_{st}-e_{st}$. Based on these values, the posterior distribution P(D,R|A) for the layered model can be represented by Equation (7):

(7) $$P(D,R|A)\propto R_{s}^{Y_{s}}{\left(1-R_{s}\right)}^{N_{s}}const_{layered}$$

We represent the connections of blocks in other layers as $R_{\neg s}=(R_{1},R_{2},\ldots ,R_{s-1},R_{s+1},\ldots ,R_{l})$. This notation allows us to express the distribution P(R|D,A) as $P(R_{1},R_{2},\ldots ,R_{l}|D,A)$. Establishing the connections between ${block}_{s}$ and other blocks, P(D,R|A) can be represented by Equation (8). Because $P\left(R|D,A\right)\propto P(A|D,R)P(R)$, the proportionality in Equation (8) allows the term $P(R_{\neg s}|D,A)$ to be eliminated. This elimination ultimately leads to Equation (9).

(8) $$P(R|D,A)=P(R_{s}|D,A,R_{\neg s})P(R_{\neg s}|D,A)$$

(9) $$P(R_{s}|D,A,R_{\neg s})\propto P(A|D,R)P(R)$$

According to our proposed layered model definition, given that $P(R)\propto {const}_{layered}$ and $P(A|D,R)\propto R_{s}^{Y_{s}}{\left(1-R_{s}\right)}^{N_{s}}$, we can ultimately derive Equation (10). Equation (10) indicates that the block connection $R_{s}$ depends on other parameters in the layered model and satisfies $R_{s}\sim B(Y_{s}+1,N_{s}+1)$.

(10) $$P(R_{s}|D,A,R_{\neg s})\propto R_{s}^{Y_{s}}{\left(1-R_{s}\right)}^{N_{s}}const_{layered}$$

For a fixed block assignment D, new $R_{s}^{\left(t+1\right)}$(1≤s≤l) values can be sequentially obtained using Gibbs sampling. This process relies on the density variation in the distribution P(R|D,A). In the t+1-th sampling, the range of $R_{s}^{\left(t+1\right)}$ needs to be controlled. This control ensures two objectives: smooth parameter updates in the layered model and increased likelihood that updated parameters reflect the actual network structure. Because the block connections $R_{s}$ follow a beta distribution, $R_{s}^{\left(t+1\right)}$ in the (t+1)-th sampling should be controlled such that $R_{s-1}^{\left(t+1\right)}<R_{s}^{\left(t+1\right)}<R_{s+1}^{\left(t\right)}$. This restriction ensures that $R_{s}^{\left(t+1\right)}$ has a slight difference from the $R_{s}^{\left(t\right)}$ of the previous sampling round. The sampling process incorporates an acceptance probability mechanism. If newly sampled parameters differ significantly from prior values, their acceptance probability decreases, leading to likely rejection. We determine whether the sampling is within the restricted range by calculating the peak value $p_{s}^{\left(t\right)}$ of the distribution P(R|D,A) in the t-th sampling, where $p_{s}^{\left(t\right)}=\frac{Y_{s}^{\left(t\right)}+1}{Y_{s}^{\left(t\right)}+Y_{s}^{\left(t\right)}+2}$. If the sampling is within the restricted range, i.e., $R_{s-1}^{\left(t+1\right)}<p_{s}^{\left(t\right)}<R_{s+1}^{\left(t\right)},R_{s}^{\left(t+1\right)}$ can be directly sampled from the beta distribution. If the sample $R_{s}^{\left(t+1\right)}$ meets the constraints, it is accepted; otherwise, the value is rejected, and sampling continues. For samples outside the restricted range, i.e., $p_{s}^{\left(t\right)}<R_{s-1}^{\left(t+1\right)}$ or $p_{s}^{\left(t\right)}>R_{s+1}^{\left(t\right)}$, we use rejection sampling. In this case, the beta distribution can be expressed as a function, f, of the sample x, with the specific form provided in Equation (11). Here, Γ(x) represents the gamma function.

(11) $$f(x;Y_{s}^{\left(t\right)};N_{s}^{\left(t\right)})=\frac{x^{Y_{s}^{\left(t\right)}}{\left(1-x\right)}^{N_{s}^{\left(t\right)}}\Gamma (Y_{s}^{\left(t\right)}+N_{s}^{\left(t\right)}+2)}{\Gamma (Y_{s}^{\left(t\right)}+1)\Gamma (N_{s}^{\left(t\right)}+1)}$$

We define a uniform distribution, $U(x)=\frac{1}{R_{s+1}^{\left(t\right)}-R_{s-1}^{\left(t+1\right)}}$, over the range $\left[R_{s-1}^{\left(t+1\right)},R_{s+1}^{\left(t\right)}\right]$. Based on this distribution, the value V is calculated as $V={(R}_{s+1}^{\left(t\right)}-R_{s-1}^{\left(t+1\right)})\cdot max({(R}_{s+1}^{\left(t\right)},R_{s-1}^{\left(t+1\right)})$. To ensure validity, the beta distribution function f(x) must satisfy f(x)<U(x)·V. This condition determines the acceptance probability for sample x drawn from the uniform distribution U, as shown in Equation (12). Thus, moderate sampling of $R_{s}^{\left(t+1\right)}$ can be performed within the beta distribution.

(12) $$P_{accept}(x)=\frac{f(x)}{U(X)\cdot V}$$

The next step is to sample from the distribution P(D|R,A)). We use the Markov chain Monte Carlo (MCMC) method to obtain the distribution P(D|R,A) based on P(D|R,A)∝P(A|D,R)P(D). First, we randomly assign blocks to the nodes in the network. After a certain number of iterations, we randomly select a node, ${node}_{i}$, and update its block label $D_{i}$. We use random sampling to select the new block label $D_{i}^{\prime }$ for ${node}_{i}$, i.e., $P\left(D_{i}^{\prime }|D\right)=\frac{1}{l}$. By inverting $D_{i}$, a new block assignment $D^{\prime }$ is obtained. According to the Metropolis–Hastings criterion, the probability of acceptance is calculated as shown in Equation (13).

(13) $$P_{accept}(D^{\prime })=\min(1,\frac{P(D^{\prime }|R,A)}{P(D|R,A)}\frac{P(D|D^{\prime })}{P(D^{\prime }|D)})$$

Combining the two sampling processes described above, we propose the inference algorithm for the layered model (the hub-and-spoke model follows a similar procedure, with two key modifications: the constraint l=2 and adjusted connectivity rules). Algorithm 1 describes the inference process for the layered model.
**Algorithm 1** Layered Model Inference1: **procedure** $LAYEREDMODEL\left(G,T_{Gibbs},T_{MCMC}\right)$2:  $D^{\left(0\right)}\leftarrow GenerateDistribution(G)$ //Initialize Block Assignments 3:  $D^{\left(0\right)}$,$n,e,E\;\leftarrow AnalyseAndReorder(D^{\left(0\right)})$ //Reorder under constraints 4:  **for** $R_{i}\in R$ **do** 5:     $R_{i}^{\left(0\right)}\leftarrow$ SampleFromBeta(e,E,R) 6:  **end for** 7: 8:  **for** $t_{g}\leftarrow 1\ldots T_{Gibbs}$ **do** 9:     $D^{\left(t_{g}\right)}\leftarrow D^{\left(t_{g-1}\right)}$ //Initialize Gibbs Sample 10:   **for** $t_{m}\leftarrow 1\ldots T_{MCMC}$ **do** //Sample $P\left(D|R^{t_{g}-1},A\right)$ 11:     i←ChooseNode() //Random Choose Node *i* 12:     s ←ChooseBlock() //Random Choose Block *s* 13:     $D^{t}\leftarrow D^{\left(t_{g}\right)}$ 14:     $D_{i}^{t}\leftarrow s$ 15:     $n,e,E\;\leftarrow \;UpdateDistribution\left(D,D^{\prime }\right)$ //Update Assignments 16:     $P_{accept}\leftarrow \;DecideAccept\left(P\left(D^{\left(t_{g}\right)}|R,A\right),P\left({D^{\prime }}^{\left(t_{g}\right)}|R,A\right)\right)$ 17:     **if** $P_{accept}==1$ **then** 18:         $D^{\left(t_{g}\right)}\leftarrow {D^{\prime }}^{\left(t_{g}\right)}$ //Accept new Assignments 19:     **else** 20:      $\;UpdateDistribution\left(D^{\prime },D\right)$ //Revert Change 21:     **end if** 22:      **end for** 23:      for $R_{i}\in R$ do 24:      $R_{I}^{\left(t_{g}\right)}\leftarrow SampleFromBeta(e,E,R)$ //Sample $P\left(R|D^{\left(t_{g}\right)},A\right)$ 25:      **end for** 26:     **end for** 27: **end procedure**

### 3.4. Comparison and Evaluation of Core–Periphery Structures

When assessing the performance of different core–periphery partitioning methods, we need a metric to measure the differences between different partitioning results. Our goal is to evaluate the performance of methods by comparing partitioning results with a “ground truth” or “authoritative” partitioning that aligns with shared understanding.

Core–periphery partitioning outcomes are sensitive to initialization settings and parameter choices. To account for this variability, the same method must be executed multiple times to generate diverse partitioning results. These results may form a collection of outcomes rather than a single partition. In such cases, the optimal subset can be selected or multiple subsets can be randomly sampled for clustering aggregation.

We can use the variation in information (VI) metric to compare the distances between different partitioning results. Rooted in information entropy theory, VI calculates the distance between two partitions by measuring information exchange, loss, and gain. This dual perspective makes VI particularly suitable for evaluating similarities and differences in core–periphery structural partitions.

The process of comparing the core–periphery structures is as follows. Firstly, select a node, ${node}_{i}$, from the network. The probability of this node belonging to the core node set $S_{c}$ is defined as $P\left(c\right)=\frac{n_{c}}{n}$, where $n_{c}$ and n are the sizes of $S_{c}$ and the total network, respectively. Furthermore, we define a discrete random variable of length C, which selects C nodes corresponding to the number of sets in partition P. In core–periphery structures, there is only a core node set and a periphery node set, so C takes a value of 2. The entropy of the discrete random variable C is denoted as $E\left(P\right)=-\sum_{c=1}^{C}P(c)logP(c)$. It is essential to note that $E\left(P\right)$ is the entropy of the partition P, and it is a non-negative value. This entropy depends not on the absolute node count but on the relative proportions of node sets in P.

We assume two different partitions, $P_{i}$ and $P_{j}$, where the node set $S_{c}$ in $P_{i}$ corresponds to the node set $S_{c^{\prime }}$ in $P_{j}$. The joint probability distribution of ${node}_{i}$ belonging to $S_{c}$ in partition $P_{i}$ and $S_{c^{\prime }}$ in partition $P_{j}$ is denoted as $P\left(c,c^{\prime }\right)=\frac{n_{S_{c}\cap S_{c^{\prime }}}}{n}$, where $n_{S_{c}\cap S_{c^{\prime }}}$ represents the number of nodes assigned to both $S_{c}$ and $S_{c^{\prime }}$ in partitions $P_{i}$ and $P_{j}$, respectively. We use mutual information (MI) to describe the information about partition $P_{j}$ provided by partition $P_{i}$. When selecting ${node}_{i}$ in the network, the uncertainty of ${node}_{i}$ in partition $P_{i}$ is denoted as $E\left(P_{i}\right)$. If it is found that ${node}_{i}$ is assigned to any node set in $P_{j}$, the uncertainty $E\left(P_{i}\right)$ should decrease accordingly. The decrease in uncertainty is distributed among the C−selected nodes, partially explaining the principle behind MI. We use Equation (14) to express the MI between partitions $P_{i}$ and $P_{j}$.

(14) $$MI(P_{i},P_{j})=\sum_{c=1}^{C}\sum_{c^{\prime }=1}^{C^{\prime }}P(c,c^{\prime })\log\frac{P(c,c^{\prime })}{P(c)P(c^{\prime })}$$

Based on the above content, we consider $E\left(P_{i}\right)$ and $E\left(P_{j}\right)$ as measures of uncertainties for node sets in two distinct partitions. The mutual information $MI(P_{i},P_{j})$ represents the shared knowledge between these partitions, which effectively reduces uncertainty about node set assignments. To compute the variation in VI information between partitions, we first calculate the total uncertainties by summing $E\left(P_{i}\right)$ and $E\left(P_{j}\right)$. Next, we subtract the mutual information $MI\left(P_{i},P_{j}\right)$ to eliminate the influence of shared knowledge. Equation (15) provides the calculation formula for the VI distance.

(15) $$VI(P_{i},P_{j})=E(P_{i})+E(P_{j})-2MI(P_{i},P_{j})$$

**Figure 6:**
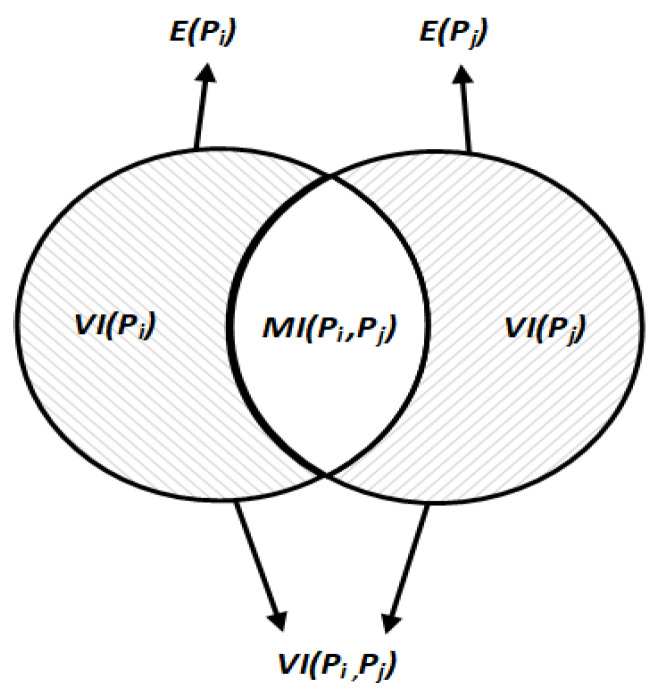
Image description of the VI distance.

Figure 6 intuitively describes the VI distance for core–periphery structures. The shaded areas represent the uncertainty components that contribute to the VI distance. The middle blank area represents the mutual information (MI) shared between the two partitions.

After determining the VI distance as a comparison metric for core–periphery structures, we found significant differences in the core–periphery structures obtained with different model parameters. Therefore, we need to evaluate each core–periphery structure further. Based on the principle of MDL, we decompose the evaluation into two components: the number of bits $L\left(M\right)$ required to describe the core–periphery stochastic block model itself and the number of bits needed for the model to describe the network data. This is formalized as ${MDL}_{M}=L\left(M\right)+L(D,R,A|M)$. The length of the model describing the network data can be approximated as $L\left(D,R,A|M\right)\approx P\left(A,D_{M}|M\right)$. This form can be further obtained by integrating over the block connection P, as shown in Equation (16).

(16) $$P(A,D_{M}|M)=\int P(D_{M}|M)P(R|M)P(A|D_{M},R,M)dR$$

Direct computation of this integral is computationally challenging. To address this, we use Monte Carlo simulation to sample n values of R from its prior distribution P(R), following the sampling process described in Section 4.2. These samples approximate the integral for the model description length. The sum of intervals among the n sampled points is 1, and the distribution of intervals is consistent and randomly combined. Therefore, the distribution of these intervals follows a Dirichlet distribution. The samples $R_{s}$ can be described by the intervals ${blank}_{i}\;$(${blank}_{i}=R_{i+1}-R_{i}$) of the samples, i.e.,$\;R_{s}=1-\sum_{i\leq s}{block}_{i}$. We simplify the calculation by applying a logarithmic transformation to the model description length. This yields the expression in Equation (17), where $MAX=max(logP(A|D_{M},R_{s},M))$.

(17) $$\log P(A,D_{M}|M)\approx MAX-\log n+\log P(D_{M}|M)+\log\sum_{i=1}^{n}e^{\log P(A|D_{M},R_{s},M)-MAX}$$

By approximating $P\left(A,D_{M}|M\right)$ and combining with the estimated model encoding lengths, we can obtain the MDL values for different core–periphery structures. For core–periphery structures $M_{i}$ and $M_{j}$, the MDL ratio is calculated as ${ratio}_{MDL}=\frac{P(A,D_{M_{i}}|M_{i})P(M_{i})}{P(A,D_{M_{j}}|M_{j})P(M_{j})}$. This ratio facilitates direct comparison between implemented structures. Since different core–periphery structures are assumed to be equiprobable, the MDL ratio depends solely on the relative likelihoods of their distributions. We apply a logarithmic transformation to the MDL ratio to quantify statistical differences. This process is formalized in Equation (18), converting the ratio into an interpretable metric.

(18) $$-\log ratio_{MDL}=-\log P(A,D_{M_{i}}|M_{i})-(-\log P(A,D_{M_{j}}|M_{j}))$$

Our comparison and evaluation framework provides two key capabilities: First, it estimates the quality of different core–periphery structures and assesses their ability to characterize network features. These evaluations inform weight settings during locally weighted core–periphery clustering, offering data-driven guidance for structural optimization.

### 3.5. Local Weighted Aggregation of Core–Periphery Structures

Ensemble learning is an essential method in machine learning that combines multiple models into a more effective one. Classifier aggregation and clustering aggregation belong to supervised and unsupervised learning, respectively. When analyzing the core–periphery structure in NFT transfer networks, we are more concerned with the network’s structural features. Therefore, we consider using clustering aggregation methods to enhance the reliability of core–periphery partitioning results and the expressive power of network features.

**Figure 7:**
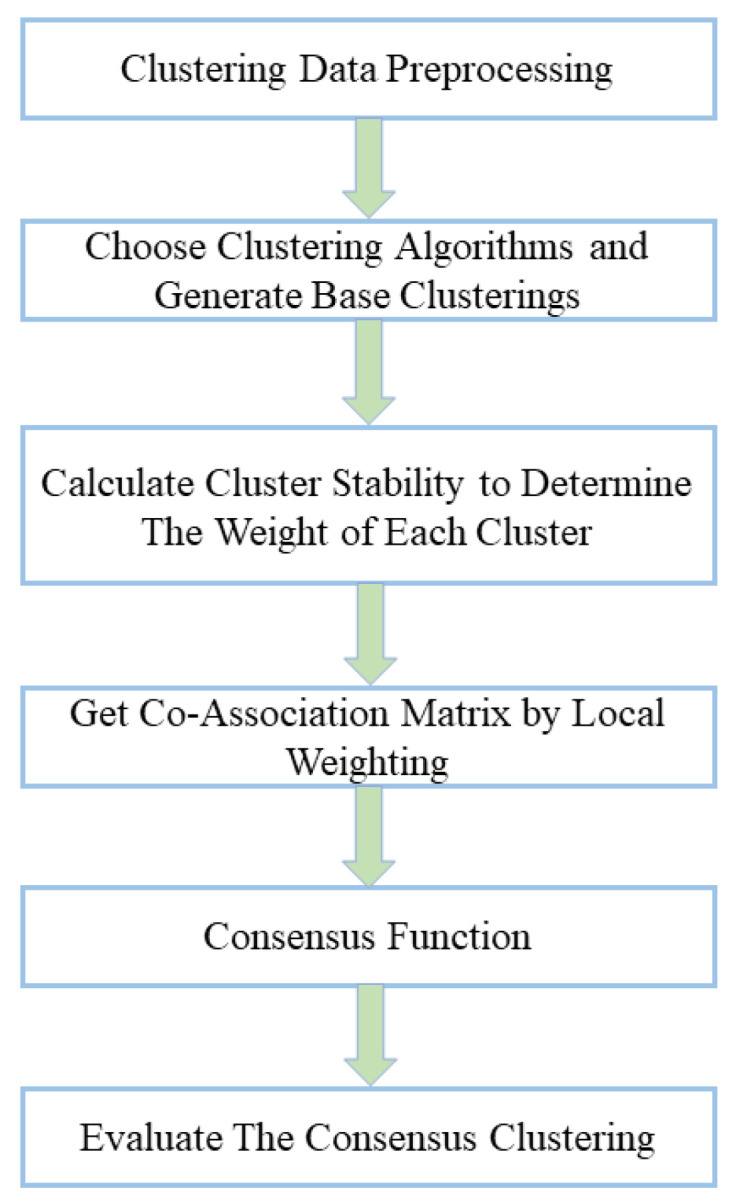
The process of weighted ensemble clustering.

Clustering aggregation enhances the robustness and accuracy of clustering results by considering the diversity of global clustering. Weighted clustering aggregation is an extension that considers the weights of different clusters in the aggregation process, thereby improving the aggregation effect. Figure 7 shows a schematic diagram of weighted clustering aggregation.

Most networks exhibit complex structures in practical applications, challenging accurate and comprehensive analysis. Clustering aggregation methods have been introduced to graph structures to address these challenges. The core idea combines multiple node partitioning results to generate improved consolidated partitioning. However, the field of graph clustering currently lacks a universally accepted definition. This conceptual ambiguity has led to the proliferation of diverse algorithms with varying aggregation processes.

Taking the analysis of core–periphery structures in networks as an example, different core–periphery partitioning methods yield distinct core–periphery structures. These different core–periphery partitioning results essentially partition the nodes in the network into different node clusters. We represent graph G as a binary data structure, $G=\left(V,E\right)$. Here, V denotes the set of nodes ($V=\left\{{node}_{1},{node}_{2},\ldots ,{node}_{n}\right\}$), and E represents the set of edges ($E=\left\{\left(source,target\right)|source,target\in V,source<target\right\}$). The partitioning results obtained through a specific core–periphery partitioning method consist of two node clusters, namely, $C_{core}$ and $C_{pi}$. Here, $C_{core}^{i}=\left\{{node}_{core}^{1},{node}_{core}^{2},\ldots ,{node}_{core}^{m}\right\}$ and $C_{pi}^{i}=\left\{{node}_{pi}^{1},{node}_{pi}^{2},\ldots ,{node}_{pi}^{n-m}\right\}$. Therefore, this type of core–periphery partitioning result can be represented as $P_{i}=\{C_{core}^{i},C_{pi}^{i}\}$. For real networks, aggregation results from multiple partitioning methods generate a composite structure $P=\{P_{1},P_{2},\ldots ,P_{n}\}$. Building upon graph aggregation theory, this study integrates weighted clustering aggregation techniques. Specifically, we unify core–periphery partitioning results within cluster P*P* to enhance structural characterization.

**Figure 8:**
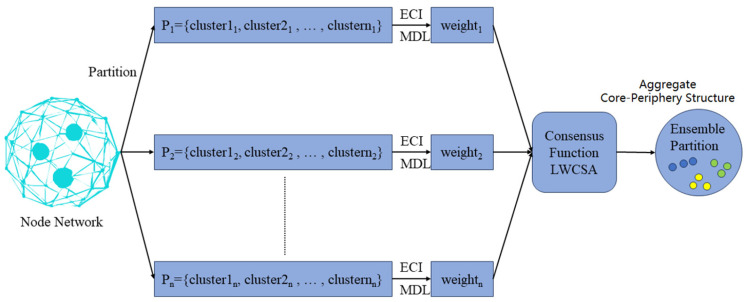
The process of LWCSA. The original node (blue green on the left) was cut by multiple partitions → ECI/MDL dynamic weighting → LWCSA consensus aggregation, and finally achieved visual separation of the three types of structures by color saturation. Core (blue nodes—network hubs, e.g., blockchain super-nodes); Transition (yellow nodes—partial core features with weak connectivity); Periphery (green nodes—network outskirts).

We introduce the locally weighted core–periphery structure aggregation (LWCSA) method, which integrates bipartite graph models to balance global diversity and local reliability. This method aims to improve the accuracy and robustness of consensus core–periphery structures. Figure 8 illustrates the process of LWCSA. The approach consists of three steps: the uncertainty estimation of node sets, the reliability testing of node sets, and core–periphery structure aggregation based on local weighted graph partitioning. Firstly, we estimate the uncertainty of each core–periphery partition using the concept of information entropy. Given a node set, $s_{i}\in S$, and a core–periphery partition, $p_{k}\in P$, where S and P represent all the node sets and core–periphery partition sets, $p_{k}=\{p_{k}^{1},p_{k}^{2},\ldots ,p_{k}^{m}\}$ and $P=\{p_{1},p_{2},\ldots ,p_{k},\ldots ,p_{M}\}$, respectively. The uncertainty of $s_{i}$ with respect to $p_{k}$ can be calculated by considering how nodes in $s_{i}$ are aggregated in $p_{k}$. Firstly, we compute the distribution $p\left(s_{i},p_{k}^{j}\right)=\frac{n_{s_{i}\cap p_{k}^{j}}}{n_{s_{i}}}$ of each node in $s_{i}$ across node sets in $p_{k}$. Through this distribution, we can further obtain the uncertainty $E^{k}\left(s_{i},p_{k}\right)=-\sum_{j=1}^{m}p(s_{i},p_{k}^{j})logp(s_{i},p_{k}^{j})$ of $s_{i}$ concerning $p_{k}$. In our uncertainty computation, we introduce the MDL of core–periphery structures, resulting in the uncertainty $E(s_{i},P)$ of $s_{i}$ concerning the core–periphery partition set P as shown in Equation (19).

(19) $$E(s_{i},P)=\sum_{k=1}^{M}\frac{MDL(p_{k})-\min(MDL)}{\max(MDL)-\min(MDL)}E^{k}(s_{i},p_{k})$$

After determining the uncertainty of each node set in the core–periphery partitions, we calculate their reliability. The ensemble-driven clustering index (ECI) is the reliability metric for each node set. Given the core–periphery partition set P and $s_{i}$, where there are M core–periphery partitions in P, the ECI of the node set $s_{i}$ is calculated as follows: $ECI\left(s_{i}\right)=e^{-\frac{E(s_{i},P)}{\delta M}}$. Here, due to the drastic influence of instability $E\left(s_{i},P\right)$ on the growth of ECI, we introduce a parameter, δ, in the denominator of the exponent to balance the effect of instability.

We propose LWCSA based on bipartite graphs, considering the core–periphery structures’ global diversity and local reliability. A bipartite graph is defined by two disjoint sets with no intra-set connections. In core–periphery partitions, the core and periphery nodes are also distinct. Therefore, bipartite graph methods are suitable for core–periphery structure aggregation.

We represent the NFT transfer network nodes as nodes within a bipartite graph. In this bipartite graph, the core and periphery nodes are sets from two distinct node groups. If there is an edge between nodes in the graph, it indicates that the nodes in the NFT transfer network belong to different sets of nodes in the bipartite graph. By integrating the bipartite graph framework with ECI metrics and MDL-based core–periphery segmentation, our method achieves two objectives: (1) capturing node set affiliations in the network and (2) synthesizing local reliability metrics during structural aggregation. We define the bipartite graph $G_{B}^{NFT}=(V,W)$, where V=N∪S,N represents all nodes and W represents the weight matrix of edges between two different node sets in $G_{B}^{NFT}$,$\;W=\{w_{11},w_{12},\ldots ,w_{ij},\ldots ,w_{mn}\}$. For example, the definition of edge weights $v_{i}$ and $v_{j}$ belonging to different sets is shown in Equation (20).

(20) $$W_{ij}=\left\{\begin{array}{l} ECI(v_{i}),\;\mathrm{if}\;v_{i}\in S,v_{j}\in N,v_{j}\in v_{i}\\ ECI(v_{j}),\;\mathrm{if}\;v_{j}\in S,v_{i}\in N,v_{i}\in v_{j}\\ 0,\;\;\;\;\;otherwise \end{array}\right.$$

Based on the above, we define the bipartite graph $G_{B}^{NFT}$. The next step is to partition $G_{B}^{NFT}$ into disjoint sets of nodes. We employ the spectral partitioning algorithm (SPEC) proposed by Ng et al. [18] to achieve the partitioning of $G_{B}^{NFT}$. SPEC embeds the nodes of $G_{B}^{NFT}$ into a *k-dimensional* space and then performs clustering in the *k-dimensional* space, where *k* represents the number of clusters in $G_{B}^{NFT}$. The specific process is as follows: SPEC first computes the degree matrix D of the nodes in $G_{B}^{NFT}$, where the elements of the matrix are denoted as $D\left(v_{i}\right)=\sum_{v_{j}}W_{ij}$. SPEC then calculates the normalized weighted matrix $NW=D^{-1}W$ based on the degree matrix D and the weight matrix W and identifies the top k eigenvectors to form the feature matrix ${NW}_{F}=\{f_{1},f_{2},\ldots ,f_{k}\}$. Finally, SPEC normalizes each row of ${NW}_{F}$ to unit length, resulting in k-dimensional embeddings for each node in $G_{B}^{NFT}$, which are then clustered using K-means. Nodes clustered in the same segment in the clustering result can be regarded as belonging to the same set, thus obtaining the partitioning result of $G_{B}^{NFT}$. The partitioning result of $G_{B}^{NFT}$ corresponds to the final aggregated core–periphery structure. Algorithm 2 outlines the LWCSA scheme.
**Algorithm 2** Locally Weighted Core-periphery Structure Aggregation  **Input:** the set of core periphery partitions $P\left(p_{1},p_{2},\ldots p_{n}\right)$.1: Compute the uncertainty of the sets of nodes in P. 2: Compute the model description length of each structure. 3: Combine model description length to compute the ECI index of sets of nodes in *P.* 4: Build the bipartite graph based on Citation network. 5: Partition the graph into different part. 6: Group the nodes in the same part into one set and get all sets of Citation network. 7: Get the consensus core periphery structure through the obtained sets. **Output:** the consensus core periphery structure $p^{*}$.

## 4. Experimental Analysis

In this section, we process several mainstream NFT token transaction datasets and construct NFT transfer networks. We apply the proposed core–periphery stochastic block model to the NFT transfer networks for experimentation. Additionally, we compare the proposed core–periphery structure aggregation method with other clustering aggregation methods.

Our experiments were conducted on a machine with an Intel(R) Core(TM) i7-12490F CPU @2.90 GHz and 16 GB of RAM.

### 4.1. NFT Transaction Dataset and Evaluation Metrics

Our experiments utilized ten NFT transaction record datasets obtained from the Etherscan platform. These datasets were processed to extract the corresponding data. The ten datasets are as follows: the gaming token dataset Age of Dino (https://etherscan.io/nft-top-contracts, accessed on 26 February 2024); the virtual fashion dataset ChuBBiT Official (https://etherscan.io/nft-top-contracts); the NFT digital art projects datasets HashMasks (https://github.com/epfl-scistimm/2021-IEEE-Blockchain, accessed on 26 February 2024) and Art Blocks (https://github.com/epfl-scistimm/2021-IEEE-Blockchain); the copyright image project datasets Cryptopunks (https://github.com/epfl-scistimm/2021-IEEE-Blockchain), Bored Ape Yacht Club (https://github.com/epfl-scistimm/2021-IEEE-Blockchain), and MoonCats (https://github.com/epfl-scistimm/2021-IEEE-Blockchain); and the metaverse datasets CryptoVoxels (https://github.com/epfl-scistimm/2021-IEEE-Blockchain), Decentraland (https://github.com/epfl-scistimm/2021-IEEE-Blockchain), and Meebits (https://github.com/epfl-scistimm/2021-IEEE-Blockchain). The selected NFT platforms exhibit high transaction volumes, representing dominant market patterns. Our datasets span multiple NFT sectors, capturing diverse transaction behaviors. This cross-domain sampling ensures experimental results exhibit strong representativeness and comprehensiveness. Detailed platform metadata (transaction metrics, sector classification, etc.) are systematically cataloged in Table 1.

Based on our research, Table 1 lists the established times of transactions and the innovative contract addresses of the ten datasets corresponding NFT platforms. Table 2 elaborates on the total assets, number of NFT holders, and NFT transfer quantities for these NFT projects or platforms. The data show that most of the selected NFT platforms were established early and already possess significant transaction volumes and market shares. Some newer NFT platforms still have the potential for transaction volume expansion. These survey results indicate that our selected datasets were scientifically and effectively chosen. Finally, Table 3 presents the network characteristic information for these ten datasets, where the abbreviations correspond to the dataset names DIN, CBT, HM, AB, CP, BAYC, MC, CV, DT, and MBT.

The two most widely used quality evaluation metrics for clustering partitions are the normalized mutual information (NMI) and the adjusted Rand index (ARI). The larger the values of these two indices, the more likely the clustering partition results will be reliable. Therefore, in our experiments, we used the NMI and ARI indices to evaluate the quality of the core–periphery partition results.

The NMI index quantifies the shared informational content between two clustering partitions. It is a standard metric for evaluating the similarity between derived clusterings and ground truth partitions. Given $C^{\prime }$ as the obtained clustering partition and $C^{G}$ as the ground truth clustering partition, the NMI index between these two clustering partitions can be computed using Equation (20). Here,$\;n_{ij}$ represents the number of elements shared between clustering $C_{i}^{\prime }$ in $C^{\prime }$ and clustering $C_{j}^{G}$ in $C^{G}$,$\;n^{\prime }$ denotes the total number of clusters in $C^{\prime }$, and $n^{G}$ represents the total number of clusters in $C^{G}$.

(21) $$NMI(C^{\prime },C^{G})=\frac{\sum_{i=1}^{n^{\prime }}\sum_{j=1}^{n^{G}}n_{ij}\log\frac{n_{ij}n}{n_{i}^{\prime }n_{j}^{G}}}{\sqrt{\sum_{i=1}^{n^{\prime }}n_{i}^{\prime }\log\frac{n_{i}^{\prime }}{n}\sum_{j=1}^{n^{G}}n_{j}^{G}\log\frac{n_{j}^{G}}{n}}}$$

The adjusted Rand index (ARI) is an enhanced version of the Rand index (RI). It quantifies the agreement between clustering results and ground truth labels. While computationally straightforward, the RI may overestimate clustering quality in specific scenarios. The ARI addresses this limitation by incorporating random allocation adjustments. This modification expands the metric’s range, enabling a more comprehensive partition evaluation. Unlike the normalized mutual information (NMI) index, the ARI accounts for two critical factors: similarity between clustering results and ground truth, and random allocation effects. The ARI can be calculated using Equation (21). Here, $O_{YY}$ represents the number of pairs of elements that belong to the same clusters in both $C^{\prime }$ and $C^{G}$,$\;O_{YN}$ represents the number of pairs of elements that belong to the same clusters in $C^{\prime }$ but different clusters in $C^{G}$, $O_{NY}$ represents the number of pairs of elements that belong to other clusters in $C^{\prime }$ but the same clusters in $C^{G}$, and $O_{NN}$ represents the number of pairs of elements that belong to different clusters in both $C^{\prime }$ and $C^{G}$.

(22) $$ARI(C^{\prime },C^{G})=\frac{2(O_{NN}O_{YY}-O_{YN}O_{NY})}{\left(O_{NN}+O_{NY})(O_{NY}+O_{YY})+(O_{NN}+O_{YN})(O_{YN}+O_{YY}\right)}$$

We conducted multiple experiments across ten NFT datasets to evaluate the final aggregated core–periphery structure. These experiments employed varied parameter settings and distinct core–periphery stochastic block models. We constructed a partition set containing numerous base core–periphery structures. The number of objects in each core–periphery partition set was determined based on the specific number of nodes and edges in the dataset being used. After obtaining the core–periphery partition set, to ensure that the base core–periphery structures selected for each round of core–periphery structure aggregation were random, we randomly selected twenty base core–periphery structures from the set.

### 4.2. Extraction and Evaluation of Core–Periphery Structures

To obtain the core–periphery structure partition sets required for core–periphery structure aggregation and the corresponding MDL values for each core–periphery structure, we conducted experiments to extract and evaluate core–periphery structures. This process generated the required partition sets and MDL values for aggregation. The experiments utilized two core–periphery stochastic block models (hub-and-spoke and LAYERED) across ten datasets. MDL values were computed based on the extracted structures.

The hub-and-spoke model includes a Gibbs sampling count parameter and fixes the layer number at two. In contrast, the layered model allows adjustments to both sampling iterations and layer counts. By tuning these parameters, we derived diverse core–periphery structures. MDL optimization was then applied to select statistically superior configurations.

Table 4 details the structures obtained using the following metrics: model type, Gibbs iterations, layer count, core quantity, core node proportion, and average MDL per node. Bold entries indicate the optimal core–periphery structures.

We calculated the overlap ratio between core nodes identified by the hub and layered models for deeper analysis. The ratio is computed as

(23) $$O\mathrm{ver}lapRatio=\frac{\left|{Nodes}_{Hub}\cap {Nodes}_{Layered}\right|}{\left|{Nodes}_{Hub}\cup {Nodes}_{Layered}\right|}$$

Here is the revised version with shortened sentences while retaining all technical terms and logical flow: The results demonstrate strong alignment between the two models. Over 87% of core nodes identified by the hub model are consistently detected by the layered model. This high overlap confirms substantial methodological agreement. However, the layered model provides enhanced structural resolution. It identifies intermediate nodes that bridge core and peripheral layers—a feature absent in the hub framework. This granular detection enables the layered model to characterize static NFT transport network topologies better. It precisely captures nuanced naming patterns in the network architecture that the hub model overlooks.

Additionally, we computed the VI distance to measure the differences between different core–periphery structures within the same dataset. Table 5 presents the VI distances calculated for the core–periphery structures. Based on these differences, we further aggregated different core–periphery structures.

### 4.3. Determination of the Balancing Parameter δ

Our core–periphery aggregation scheme introduces a balancing parameter, δ, to control the influence of node set uncertainty during aggregation. This parameter is integrated into the ensemble-driven clustering index (ECI) (see Section 3.5). Adjusting δ modulates the impact of instability, improving aggregation performance. We tested 20 randomly selected core–periphery partition sets across datasets to evaluate delta values. We ran the aggregation method for each dataset 50 times with varying δ values. Performance was assessed using the average normalized mutual information (NMI) index. Table 6 shows the NMI values of aggregated structures for different δ. The results indicate minimal NMI variation after applying δ, suggesting weakened uncertainty effects. Empirical analysis reveals that δ=0.2 delivers optimal aggregation outcomes in most cases. Thus, we fixed δ=0.2 for all experiments.

### 4.4. Comparison Between Aggregated Core–Periphery Structures and Base Core–Periphery Structures

**Figure 9:**
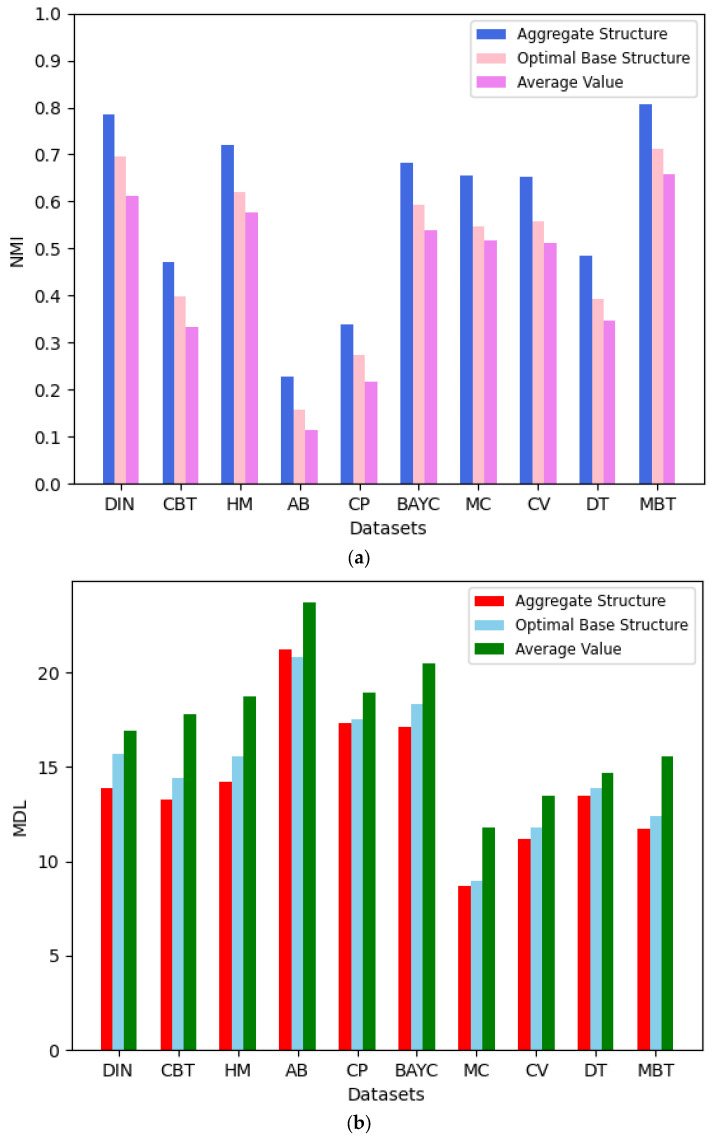
(**a**) Average performance in terms of NMI of our method; base structure over 100 runs. (**b**) Average performance in terms of MDL of our method; base structure over 100 runs.

Core–periphery structure aggregation aims to derive more effective and representative configurations. This is achieved by balancing partition diversity and local result reliability. To evaluate aggregated versus base structures, we compare their network characteristics and aggregation effects using two metrics: the NMI index and MDL value. For each dataset, we executed the LWCSA method 100 times. Each iteration randomly selected base core–periphery structures for aggregation. Finally, we obtained the NMI index and MDL value of the aggregated core–periphery structure, the optimal basic core–periphery structure, and the average NMI index and MDL value of the basic core–periphery structure, as shown in Figure 9.

The figure shows that using our proposed local weighted core–periphery structure aggregation method results in a better model representation and more accurate reference significance compared to the basic core–periphery structures. Although, in a few cases, the MDL value of the aggregated core–periphery structure may be lower than that of the optimal basic core–periphery structure, it still achieves a good aggregation effect. Therefore, the core–periphery structure aggregation method is effective and more advantageous.

### 4.5. Comparison of Our Method with Other Aggregation Methods

In this section, we compare LWCSA with other aggregation methods. We use the NMI index and ARI as evaluation metrics to assess the performance of the aggregated core–periphery structures obtained using different aggregation methods. We compare our method with Huang et al.’s local weighted evidence accumulation (LWEA) method [19], Strehl et al.’s clustering similarity partitioning algorithm (CSPA) [20], and Li et al.’s non-negative matrix factorization (NMF)-based consensus clustering method [21].

**Figure 10:**
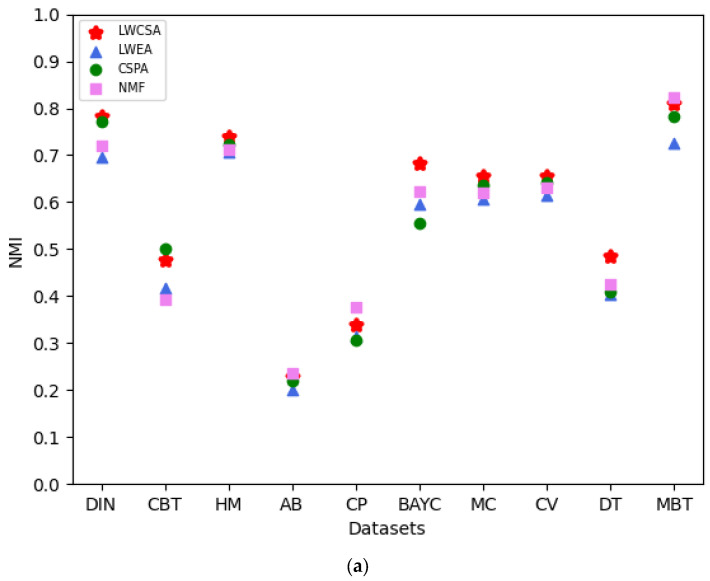

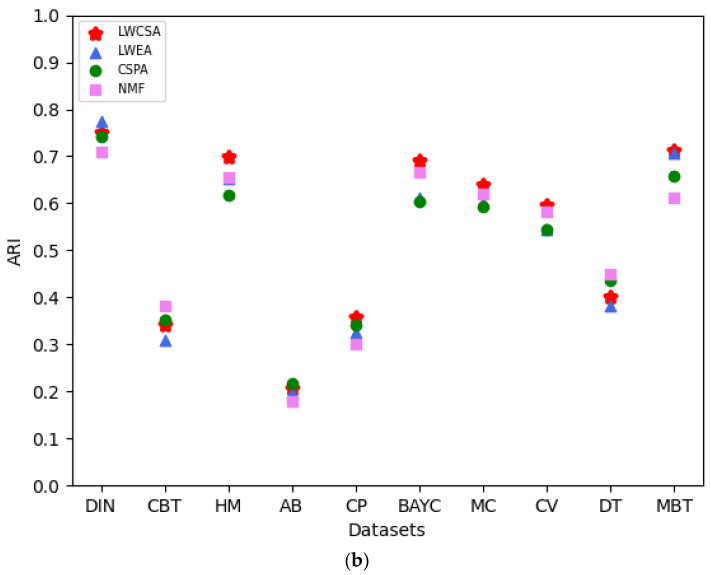
(**a**) Comparison of the aggregation performance of our method with that of other methods based on NMI. (**b**) Comparison of our method’s aggregation performance with other methods based on ARI.

We conducted 100 core–periphery structure aggregation processes to ensure fair comparisons on each of the ten datasets. Finally, by comparing the NMI indices and ARIs of the aggregated core–periphery structures obtained using various aggregation methods, as shown in Figure 10, we found that our method outperforms the others. The NMI index and ARI assess the correlation and consistency between the aggregated core–periphery structures and the accurate core–periphery node sets of the network, respectively. As shown in the figure, our method exhibits a specific improvement in the NMI index and ARI compared to other methods on most datasets, with the improvements ranging from 2.6% to 7.1%. Therefore, using our method for core–periphery structure aggregation in analyzing NFT transfer networks has advantages.

### 4.6. The Aggregation Effect of the Core–Periphery Structure and the Network Analysis Results

**Figure 11:**
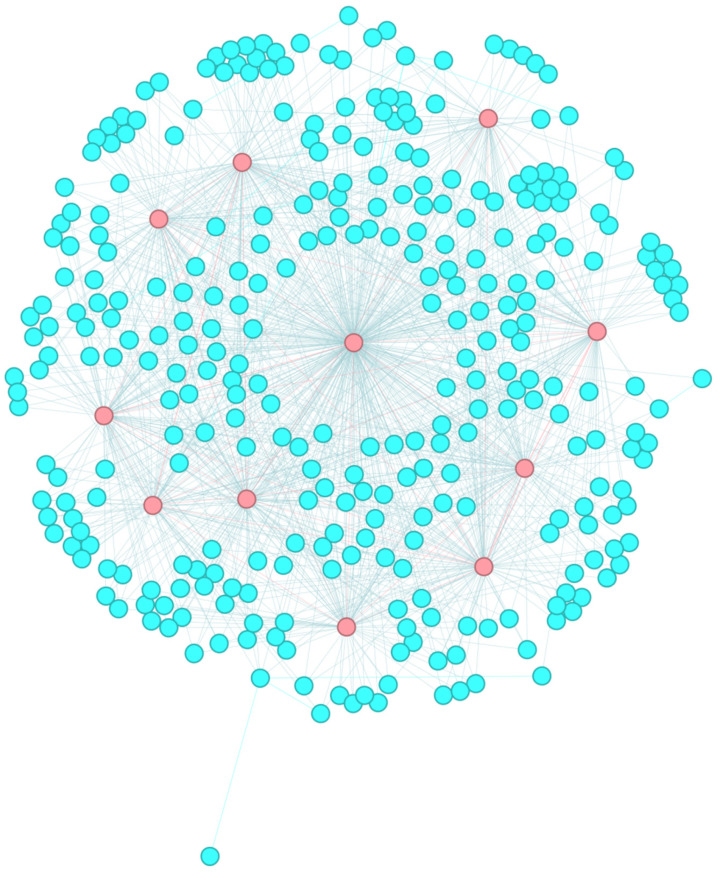
The core–periphery structure of CBT (core node set (red) and peripheral node set (blue)).

We employed a locally weighted aggregation method for core–periphery structures to obtain more efficient aggregated core–periphery structures. Using the network visualization tool Gephi (https://gephi.org/), we depicted the NFT transfer networks of ten datasets after data processing, highlighting core nodes based on the aggregated core–periphery structures. Taking the CBT dataset as an example, the corresponding network graph is shown in Figure 11. We observed that each NFT transfer network is distinctly partitioned into a core node set (highlighted in red) and a peripheral node set (highlighted in blue), with denser connections among core nodes and sparser connections among peripheral nodes. This further demonstrates the effectiveness of our core–periphery structure aggregation process.

We applied the aggregated core–periphery structure to further analyze the NFT trading market. The experimental results reveal that core nodes constitute 2.3–5% of all nodes in NFT transfer networks. This proportion is significantly smaller than the share held by dominant NFT traders in real-world markets. Core nodes mainly consist of large NFT platforms, NFT digital asset portfolios, and other emerging NFT projects, while individual NFT traders occupy a smaller portion.

The comprehensive analysis shows that the NFT trading market is highly concentrated. This demonstrates that a small number of major traders control the majority of NFT transactions and token circulation. Although there are many individual NFT traders, their trading volume is relatively low. This may lead to a monopolistic situation in NFT trading and high trade barriers, making it difficult for new entrants to become core nodes.

## 5. Conclusions

This study comprehensively analyzes the NFT transfer network, revealing key characteristics of its core–periphery structure and transaction patterns. We propose a Bayesian stochastic block model-based evaluation method and a structural aggregation scheme to address inconsistencies in core–periphery partitioning. Integrating these approaches enhances the reliability and robustness of partitioning results, enabling precise characterization of the network’s structural features. These findings provide novel insights into the internal organization of NFT markets and advance methodological frameworks for related research. The proposed framework also supports anomaly detection in NFT transactions and informs broader blockchain technology applications.

The experimental results demonstrate a distinct core–periphery structure in the NFT transfer network. Core nodes predominantly represent established digital art platforms and large-scale NFT holders, while peripheral nodes comprise less-active individual investors and small-scale platforms. This structural pattern deepens understanding of participant dynamics in NFT markets and facilitates targeted regulatory design. Notably, core nodes embody key traders who act as pivotal market influencers. Analysis of their transaction records offers critical insights into market mechanisms and evolutionary trends, providing actionable decision-making guidance for investors and trading platforms.

## Figures and Tables

**Table 1:** NFT project information.

Project/Platform	Domain	Established Time	Contract Address
DIN	Gaming token	January 2024	0xf4ecc1c74d120649f6598c7a217abaffdf76cd4f
CBT	Virtual Wearness	August 2023	0x76603b2dc8e75fd3411935169ac30f6b3387b7a2
HM	Digital art	January 2021	0xc2c747e0f7004f9e8817db2ca4997657a7746928
AB	Digital art	July 2018	0xa7d8d9ef8d8ce8992df33d8b8cf4aebabd5bd270
CP	Copyright image	January 2017	0xb47e3cd837ddf8e4c57f05d70ab865de6e193bbb
BAYC	Copyright image	April 2021	0xbc4ca0eda7647a8ab7c2061c2e118a18a936f13d
MC	Copyright image	August 2023	0xc3f733ca98e0dad0386979eb96fb1722a1a05e69
CV	Metaverse	January 2018	0xa58b5224e2fd94020cb2837231b2b0e4247301a6
DT	Metaverse	March 2018	0x102daabd1e9d294d4436ec4c521dce7b1f15499e
MBT	Metaverse	May 2021	0x7Bd29408f11D2bFC23c34f18275bBf23bB716B

**Table 2:** Transaction size information of the NFT platform.

Project/Platform	Asset	Owners	Trade Volume
DIN	3459ETH	2358	11,319
CBT	825ETH	342	2467
HM	43456ETH	5214	74,622
AB	198047ETH	38,172	591,197
CP	1236157ETH	3601	59,376
BAYC	1513201ETH	5316	277,653
MC	22050ETH	5522	70,458
CV	25207ETH	3321	17,492
DT	41245ETH	8254	222,048
MBT	181340ETH	6447	117,644

**Table 3:** NFT transfer dataset information.

Project/Platform	Nodes	Edges	Average Degree
DIN	4064	10,011	4.9
CBT	331	1193	7.2
HM	7731	17,758	4.6
AB	9592	29,259	6.1
CP	4632	12,717	5.5
BAYC	7215	18,362	5.1
MC	2046	3178	3.1
CV	2086	4481	4.3
DT	5046	10,977	4.4
MBT	6449	11,312	3.6

**Table 4:** Evaluation of different core–periphery structures.

Dataset	Model	Gibbs	Layers	Cores	Overlap Ratio	Proportion	MDL
DIN	Hub	3500	-	109	94.5%	2.7%	16.3
	Layered	1000	3	105		2.3%	15.7
CBT	Hub	500	-	11	100%	3.3%	14.4
	Layered	1000	3	11		3.3%	17.2
HM	Hub	3500	-	302	87.1%	3.9%	15.6
	Layered	4500	7	263		3.4%	18.2
AB	Hub	1000	-	433	92.4%	4.5%	20.8
	Layered	1000	7	401		4.2%	23.3
CP	Hub	5500	-	229	90.9%	4.9%	17.5
	Layered	4500	7	210		4.5%	18.1
BAYC	Hub	4500	-	287	94.1%	4.0%	18.3
	Layered	3500	7	272		3.8%	19.7
MC	Hub	2500	-	62	88.9%	3.0%	9.0
	Layered	500	6	57		2.8%	11.3
CV	Hub	4500	-	95	89.6%	4.6%	13.0
	Layered	3500	7	87		4.2%	11.8
DT	Hub	1000	-	223	96.7%	4.4%	14.2
	Layered	5000	6	218		4.3%	13.9
MBT	Hub	6000	-	204	97.1%	3.2%	12.4
	Layered	3500	5	210		3.3%	14.5

**Table 5:** Differences in the structure of the different core–periphery structures described by VI distance.

Dataset	Min VI Distance	Max VI Distance	Average
DIN	0.047	0.375	0.266
CBT	0.031	0.203	0.148
HM	0.039	0.244	0.121
AB	0.065	0.169	0.116
CP	0.045	0.313	0.162
BAYC	0.078	0.336	0.142
MC	0.028	0.201	0.127
CV	0.026	0.115	0.062
DT	0.043	0.270	0.133
MBT	0.024	0.209	0.097

**Table 6:** Core–periphery structure aggregation effects under different δ. The bold part is the optimal aggregate result for each data set.

Dataset	δ						
	0.1	0.2	0.3	0.4	0.5	1	2
DIN	0.779	**0.782**	0.775	0.751	0.742	0.729	0.710
CBT	0.445	**0.476**	0.464	0.462	0.455	0.427	0.421
HM	0.701	0.722	0.719	**0.739**	0.737	0.725	0.736
AB	**0.228**	0.222	0.218	0.220	0.224	0.207	0.203
CP	0.329	**0.339**	0.335	0.328	0.327	0.284	0.277
BAYC	0.670	**0.683**	0.679	0.681	0.653	0.641	0.647
MC	0.645	**0.655**	0.644	0.631	0.625	0.640	0.651
CV	0.628	0.648	**0.656**	0.641	0.629	0.630	0.613
DT	0.464	**0.486**	0.467	0.475	0.481	0.463	0.472
MBT	0.782	**0.809**	0.805	0.803	0.795	0.791	0.780

## Data Availability

Data will be made available on request.
